# Effect of dietary pattern on pregnant women with gestational diabetes mellitus and its clinical significance

**DOI:** 10.1515/biol-2022-0006

**Published:** 2022-03-19

**Authors:** Jianping Wang, Zuoliang Xie, Peipei Chen, Yuhuan Wang, Baoqing Li, Fen Dai

**Affiliations:** Department of Obstetrics and Gynecology, The Second Affiliated Hospital of Wenzhou Medical University, No. 306 Hualongqiao Road, Wenzhou 325000, Zhejiang, China; Department of Clinical Laboratory, The Second Affiliated Hospital of Wenzhou Medical University, Wenzhou 325000, Zhejiang, China

**Keywords:** GDM, dietary pattern management, intestinal microbiome, inflammation

## Abstract

Gestational diabetes mellitus (GDM) is a common obstetric disease among pregnant women. This study aimed to explore the effect of dietary pattern management to reduce the incidence of GDM. Here, we, retrospectively, analyzed the influence of dietary patterns on the occurrence of GDM and the correlation between dietary patterns and intestinal microbiome distribution and inflammation in pregnant women. Moreover, patients were assigned to the dietary pattern management group and the nondietary pattern management group, and the effects of dietary patterns on the intestinal microbiome distribution and inflammatory factors were investigated. We found that the intestinal microbiome was changed in GDM patients compared with the healthy controls. The relative abundance of probiotics *Lactobacillus* and *Bifidobacterium* significantly decreased in patients with GDM. Moreover, compared with the control group, the expression levels of interleukin-6 and tumor necrosis factor-α were significantly increased. Furthermore, dietary pattern management led to intestinal microbiome changes in patients with GDM. In conclusion, dietary pattern management could alleviate GDM via affecting the intestinal microbiome as well as inflammatory conditions in patients.

## 1 Introduction

Gestational diabetes mellitus (GDM) is a common obstetric disease with a high incidence rate among pregnant women [1,2,3]. According to the epidemiological investigation in China, the prevalence of GDM is about 20% [4,5,6]. Unfortunately, at present, the exact pathogenesis of GDM has not been fully elucidated, and in some cases, GDM can progress to type 2 diabetes mellitus [2,7]. Therefore, further exploration of the underlying mechanism of the disease is of great importance for discovering new biomarkers and therapeutic targets.

Earlier studies have pointed out the potential role of the gut microbiome in different diseases [8,9,10]. For example, intestinal dysbacteriosis means the abnormal changes of the intestinal microbiome might result in aberrant immune response in the colorectal tissue, which further induces the chronic inflammatory condition and consequentially induces the epithelial–mesenchymal transition of the epithelial cells and finally leads to the incidence of different disease. The roles of the gut microbiome in GDM have also been discussed previously [11,12]. Therefore, alleviating the intestinal dysbacteriosis condition may be an alternative approach for treating different GDM.

However, the effect of dietary patterns on GDM has also been discussed previously [13,14]. It was found that smart dietary patterns and vegetable dietary patterns had a protective effect against GDM and could reduce the risk of GDM [15,16]. The above two dietary patterns are rich in dietary fiber and trace elements, reducing glucose absorption and plasma insulin level and improving pancreatic function Island element sensitivity, reducing the production of reactive oxygen species, and reducing the inflammatory condition. Zareei et al. [17] studied the correlation between dietary status and GDM in 208 pregnant women at 24–28 weeks of gestation through a food frequency questionnaire. The results showed that a healthy diet rich in fruits, vegetables, and low-fat dairy products could effectively reduce the risk of GDM. At the same time, other studies [18,19,20] also confirmed that excessive intake of refined cereals, lipids, sugars compared to a small amount of fruits and vegetables before and during pregnancy will increase the incidence of GDM, suggesting that adverse dietary patterns and lifestyle can affect the production of intestinal flora and inflammatory factors, such as C-reactive protein, thus promoting the rise of fasting blood glucose and abnormal glucose tolerance.

The purpose of this study is to explore good dietary pattern management to reduce the incidence of GDM, adjust the distribution of intestinal microbiome, protect intestinal mucosa, reduce inflammatory exudation, and reduce insulin resistance, therefore, to find a new way to treat GDM and reduce the risk of GDM to mothers and infants.

## 2 Materials and methods

### 2.1 Patients

From January 2019 to May 2020, GDM pregnant women (*n* = 54) with a gestational period of 24–28 weeks and healthy controls (*n* = 117) in the obstetrics department of the Department of Obstetrics and Gynecology, the Second Affiliated Hospital of Wenzhou Medical University were enrolled in the present study. Inclusion criteria were (1) pregnant women meeting GDM diagnostic criteria (edited by Lejie, 8th edn.) and (2) age: 18–40 years old; exclusion criteria: (1) multiple pregnancies, (2) history of hypertension and diabetes, (3) other complications during pregnancy, such as polyhydramnios, oligohydramnios, and preeclampsia, (4) patients with infectious diseases, (5) taking hormone drugs before screening, and (6) unable to cooperate. GDM pregnant women were randomly allocated into two groups, and the specific randomized assignment was completed by medical statistics professionals. According to the results of sample size estimation, the subjects were randomly assigned into two groups by simple randomization (by random number generator using SPSS). The experimental group and the control group, the dietary pattern management group (the dietary pattern extracted from the pregnant women meeting GDM diagnostic criteria), and the nondietary pattern management group. The dietary pattern management group included in this study refers to that pregnant women have visited the nutrition department clinic at least twice after determining GDM. Therefore, under the guidance of professionals, they can follow it well. The balanced dietary structure is as follows: carbohydrates account for 50–60%, appropriately increasing protein intake accounts for 20–30%, and controlling fat intake accounts for 15–20%.


**Informed consent:** Informed consent has been obtained from all individuals included in this study.
**Ethical approval:** The research related to human use has been complied with all the relevant national regulations, institutional policies, and in accordance with the tenets of the Helsinki Declaration and has been approved by the authors’ institutional review board or equivalent committee.

### 2.2 Sample collection and calculation of the relative abundance of the fecal bacteria

An appropriate amount of fresh feces of the objects were collected and sent for inspection within half an hour. A total of 0.1 g fecal sample and 0.9 mL normal saline were added into a disposable centrifuge tube for mixing, and after mixing, they were diluted in series. The relative abundance of the fecal bacteria in stool samples has been calculated by quantitative polymerase chain reaction (qPCR) methods. Briefly, RNAs were isolated from the stool samples, and PCR was performed using the qPCR Green Master Mix kit (Takara, Dalian, China). The thermocycling conditions have been set as 95°C for 3 min, followed by 40 cycles of 95°C for 15 s, and 72°C for 30 s. Relative abundance of the bacteria were calculated as follows:

(\text{DNA concentration}\hspace{.25em}\text{(ng/μL)}\times \text{[}6.022\times {10}^{23}\text{]})\text{/}\hspace{2em}\text{(Length}\hspace{.5em}\text{of}\hspace{.5em}\text{template}\hspace{.25em}\text{(bp)}\times {[}1\times {10}^{9}]\times 650).]

### 2.3 Enzyme linked immunosorbent (ELISA) assay

The expression levels of interleukin-6 (IL-6) and tumor necrosis factor-α (TNF-α) were examined by ELISA method. The ELISA was conducted with the kits purchased from Beyotime (Shanghai, China). All procedures were strictly followed by the protocol of the manufacturer.

### 2.4 Statistical analysis

All experiments were carried out in triplicate. All data were analyzed using SPSS 19.0 software (SPSS Inc., Chicago IL, USA). The values were presented as mean ± SD. Statistical significance was analyzed using unpaired student’s *t*-test. *p*-value of less than 0.05 was considered statistically significant.

## 3 Results

### 3.1 Demographic characteristic of the patients and controls

The demographic characteristic of the patients and controls were as follow. The age of the GDM and control group is 27.97 ± 4.09  vs 30.24 ± 5.25, *p* = 0.0005; gravidity is 2.09 ± 1.23  vs 2.17 ± 1.33, *p* > 0.05; gestational weeks is 25.80 ± 0.88  vs 25.96 ± 0.91, *p* > 0.05; and fasting blood glucose is 4.40 ± 0.32  vs 4.76 ± 0.49, *p* < 0.001.

### 3.2 Elevated expression of IL-6 and TNF-α in serum of the GDM patients

**Figure 1:**
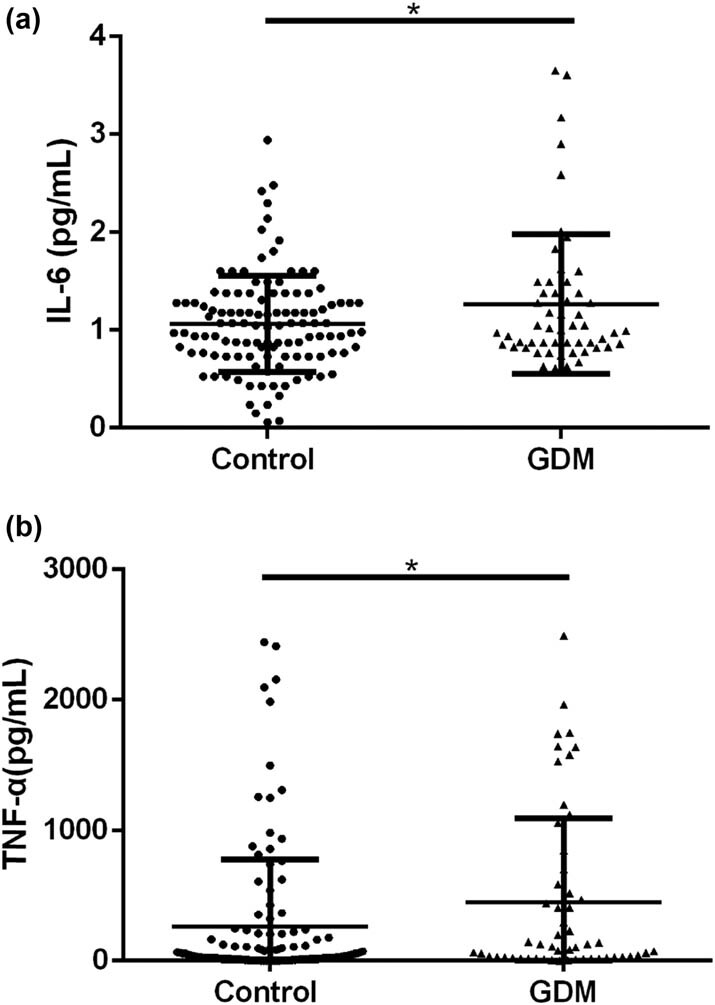
Elevated expression of IL-6 and TNF-α in serum of the GDM patients: (a) comparison of the serum level of IL-6 between control and GDM group and (b) comparison of the serum level of TNF-α between control and GDM groups. *p < 0.05, **p < 0.01.

First, the expression levels of IL-6 and TNF-α in the serum of the GDM patients and healthy controls were compared. As shown in Figure 1, compared with the control group, the expression level of IL-6 (1.062 ± 0.4897  vs 1.265 ± 0.7115, *p < 0.05) and TNF-α (260.9 ± 520.0  vs 452.0 ± 645.4, *p < 0.05) were both significantly increased.

### 3.3 Analysis of the relative abundance of probiotics in GDM and control groups

**Figure 2:**
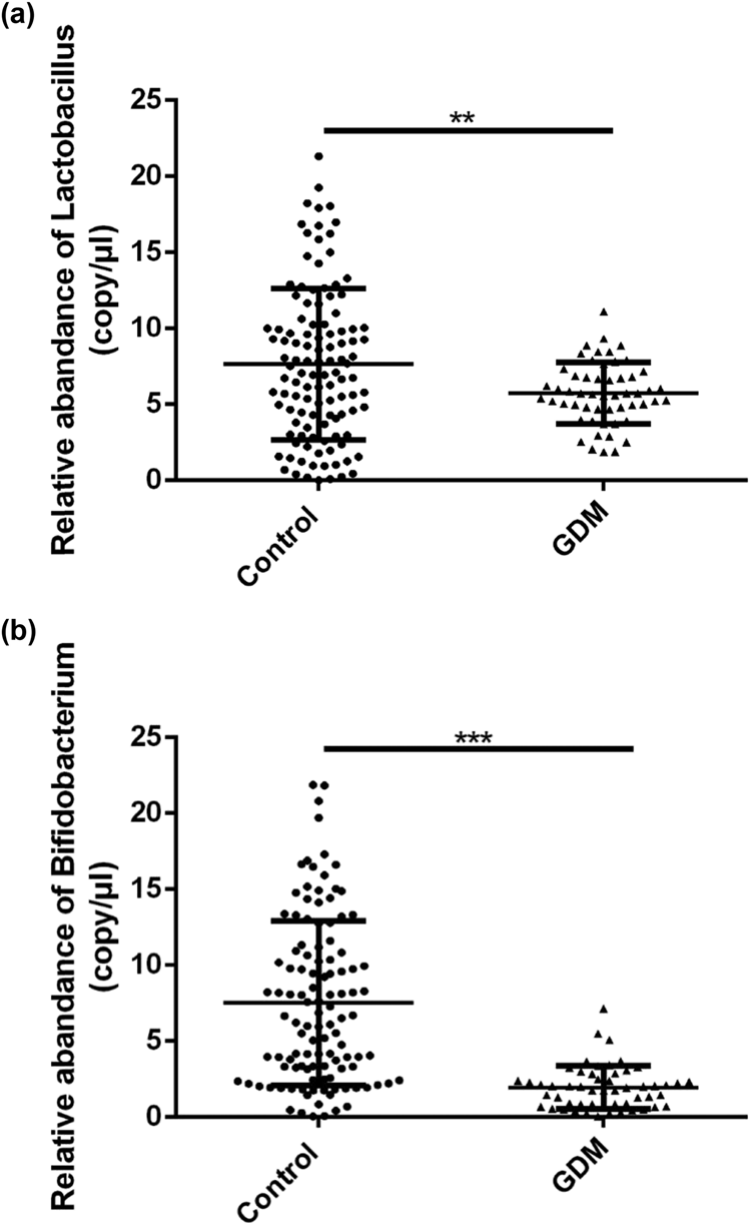
The relative abundance of *Lactobacillus* and *Bifidobacterium* in stools of patients with GDM in comparison with the controls: (a) the relative abundance of *Lactobacillus* and (b) the relative abundance of *Bifidobacterium*. **p < 0.01, ***p < 0.001.

Furthermore, the relative abundance of probiotics *Lactobacillus* and *Bifidobacterium* in the stool of the GDM and control patients were determined by real-time quantitative polymerase chain reaction (RT-qPCR) method. As shown in Figure 2, the relative abundance of *Lactobacillus* (7.643 ± 4.981  vs 5.726 ± 2.019, **p < 0.01) and *Bifidobacterium* (7.515 ± 5.421  vs 1.955 ± 1.408, ***p < 0.001) significantly decreased in patients with GDM in comparison with the controls.

### 3.4 Effect of dietary pattern on the relative abundance of fecal microbiota in GDM patients

**Figure 3:**
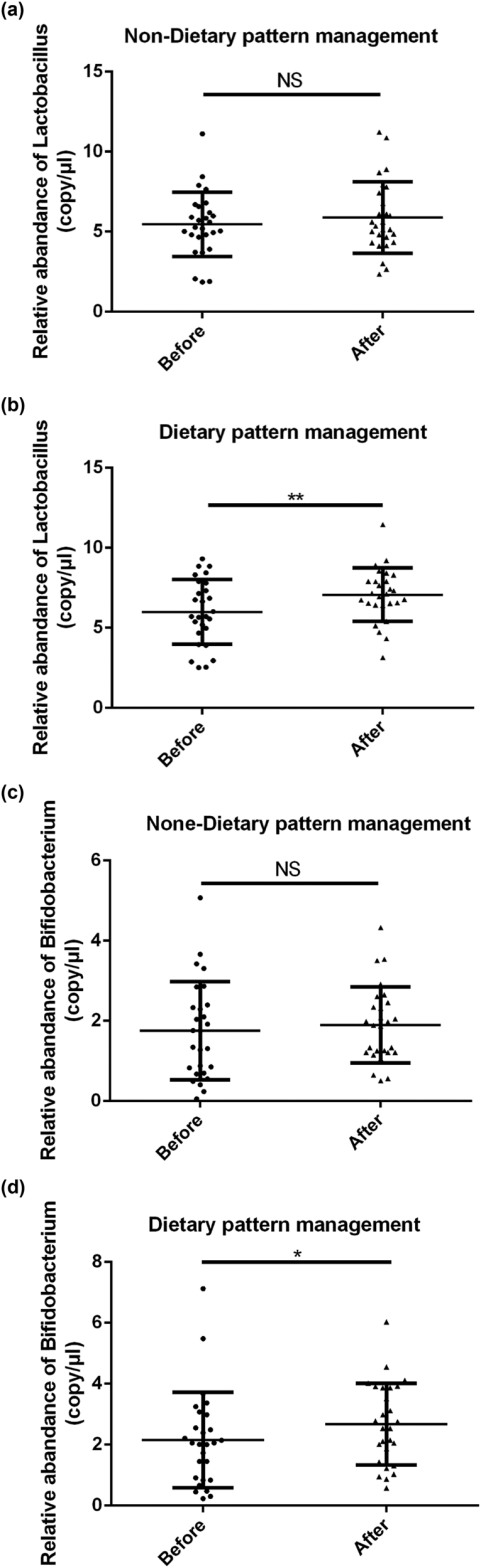
Effect of dietary pattern on the relative abundance of fecal microbiota in GDM patients: (a) the relative abundance of *Lactobacillus* and (b) the relative abundance of *Bifidobacterium*. **p < 0.01, ***p < 0.001.

Finally, the patients were randomly divided into nondietary and dietary pattern management groups, and the relative abundance of fecal microbiota was examined. As shown in Figure 3, after pattern management, the relative abundance of probiotics *Lactobacillus* (5.996 ± 2.024  vs 7.070 ± 1.667, **p < 0.01) and *Bifidobacterium* (2.156 ± 1.569  vs 2.670 ± 1.338, *p < 0.05) markedly increased, whereas the relative abundance of probiotics *Lactobacillus* (5.456 ± 2.016  vs 5.886 ± 2.238, *p* > 0.05) and *Bifidobacterium* (1.754 ± 1.222  vs 1.897 ± 0.9502, *p* > 0.05) did not significantly change in the nondietary pattern management group.

## 4 Discussion

In the present study, we focused on the roles of dietary patterns on pregnant women with GDM. We found that dietary patterns could alleviate the GDM condition by decreasing the inflammatory condition and affecting the fecal microbiota.

At present, the pathogenesis of GDM is not completely clear. The levels of insulin-antagonistic hormones such as prolactin, estrogen, progesterone, and cortisol in the placenta of pregnant women increase during pregnancy, which leads to physiological insulin resistance [21]. The increase of glucose intake and body weight of pregnant women aggravates insulin resistance during pregnancy. In recent years, studies have found that GDM is caused by the changes of hormones in pregnancy, the decrease of insulin sensitivity, and the antagonism of hormones related to insulin level regulation [22]. With the deepening of research, researchers found that the human body produces a variety of cytokines, such as TNF-α and IL-6, which play an important role in the pathogenesis of GDM. IL-6 can promote the production of TNF-α in the inflammatory response, whereas TNF-α from adipose tissue and placenta can promote insulin resistance in pregnant women, which is closely related to the pathogenesis of GDM [23,24]. In the present study, we found that the expression level of IL-6 and TNF-α were significantly increased in the GDM group, which was consistent with earlier findings, suggesting that inflammation plays a vital role in the occurrence and development of GDM.

However, studies have confirmed that excessive energy storage caused by intestinal microbiota imbalance and metabolic endotoxemia play an important role in the pathogenesis of GDM [25]. Results of earlier studies suggested that the levels of intestinal microbiota were positively correlated with the serum levels of TNF-α and IL-6. Probiotics such as *Bifidobacterium* can affect the imbalance of intestinal microbiota and prevent the occurrence of GDM [26]. In the present study, we found that the relative abundance of the probiotics *Lactobacillus* and *Bifidobacterium* were significantly decreased, which further confirmed the roles of intestinal microbiota in GDM.

Dietary pattern refers to the combination of various foods in the daily diet, including the relative composition of the types and quantity of foods. It analyzes the interaction between certain or several kinds of foods and nutrients, comprehensively evaluates the impact of dietary nutrition on disease or health, and reflects the dietary habits of different groups. The overall dietary pattern can better reflect the impact of food on disease [27,28]. Dietary pattern adjustment can regulate the distribution of intestinal flora. Good dietary patterns can promote the growth of beneficial intestinal bacteria. It can stimulate the growth of one or a few kinds of flora to have a beneficial effect on the host, protect the intestinal mucosa, and reduce inflammatory exudation. Therefore, it is widely used in the treatment of metabolic syndrome such as diabetes and obesity [29]. In current study, we found that dietary pattern management markedly increased the abundance of probiotics *Lactobacillus* and *Bifidobacterium*. Considered together, these results confirmed the protective roles of dietary pattern management in the treatment of GDM; however, the underlying mechanism still requires further investigation.

Here, we first reported the roles of dietary pattern management that might alleviate the condition of GDM via affecting the inflammatory condition and intestinal microbiota. Our results provided a new way for the management of GDM.
